# Cytokine Kinetics in Febrile Neutropenic Children: Insights on the Usefulness as Sepsis Biomarkers, Influence of Filgrastim, and Behavior of the IL-23/IL-17 Pathway

**DOI:** 10.1155/2017/8291316

**Published:** 2017-07-09

**Authors:** Orlei Ribeiro de Araujo, Reinaldo Salomão, Milena Karina Coló Brunialti, Dafne Cardoso Bourguignon da Silva, Andreza Almeida Senerchia, Fabianne Altruda de Moraes Costa Carlesse, Antonio Sergio Petrilli

**Affiliations:** ^1^Grupo de Apoio ao Adolescente e à Criança com Câncer (GRAACC), Instituto de Oncologia Pediatrica (IOP), Sao Paulo Federal University (UNIFESP), Rua Pedro de Toledo 572-Vila Clementino, 04039-001 São Paulo, SP, Brazil; ^2^Division of Infectious Diseases, Department of Medicine, Escola Paulista de Medicina, Sao Paulo Federal University (UNIFESP), Rua Pedro de Toledo 669, 10th Floor, 04039-001 São Paulo, SP, Brazil

## Abstract

**Background:**

The study aimed to describe the kinetics of various cytokines from day 1 to day 14 of the onset of fever in neutropenic children and to evaluate their performances as discriminators of sepsis in the first 24 hours of fever, the possible influence of filgrastim, and the functioning of the IL-23/IL-17 axis.

**Methods:**

IL-1*β*, TNF-*α*, IL-10, IL-12/23p40, IL-21, IL-6, IL-8, IL-17, G-CSF, and GM-CSF were measured in plasma on days 1, 2, 3, 5, and 14 from the onset of fever in 35 patients.

**Results:**

Thirteen patients (37.1%) developed sepsis. In mixed models, IL-6, IL-8, IL-10, and G-CSF showed higher estimated means in septic patients (*P* < 0.005), and IL-12/23p40 and IL-17 in nonseptic patients (*P* < 0.05). On day 1, IL-6, IL-8, and IL-10 appeared upregulated in patients who received filgrastim. Only IL-6, IL-8, IL-10, and procalcitonin were useful as discriminators of sepsis. Associating the markers with each other or to a risk assessment model improved performance.

**Conclusions:**

Cytokines kinetics showed proinflammatory and anti-inflammatory responses similar to what is described in nonneutropenic patients. IL-8, IL-6, IL-10, and procalcitonin are useful as early biomarkers of sepsis. Filgrastim upregulates expression of these markers, and we observed deficiency in the IL-23-IL-17 axis accompanying sepsis.

## 1. Introduction

The complex interactions that initiate the inflammatory response in sepsis can be triggered by molecules expressed by pathogens (PAMPs or pathogen-associated molecular patterns) or derived from the injured tissues themselves (DAMPs or damage-associated molecular patterns). Inflammatory processes that originate from bacterial infections can be similar, in terms of fever and expression of mediators, to the ones arising from traumatic tissue injury and tumors [1, 2]. Diagnosing bacterial infections in children with cancer can pose difficulties, due to inflammation already present by intermittent exposure to signaling DAMPs released from normal or malignant cells destroyed by cytotoxic treatment. These children often present signs of systemic inflammatory response syndrome (SIRS), such as fever and tachycardia in the absence of infection (sterile inflammation). However, the rapid diagnosis of infections in these patients is critical because the delay in the administration of appropriate antibiotics is associated with high mortality. Furthermore, chemotherapy is postponed in the presence of infection. Thus, differentiating sterile inflammation from infection is also important to avoid interruptions in treatment [3–5].

Information on proinflammatory and anti-inflammatory immune responses has increased in the recent years, demonstrating sepsis as a highly dynamic biological process, with immunosuppression signals coexisting with the inflammation from the initial stages [6]. One of the immunosuppressive mechanisms is deficient production of inflammatory cytokines, such as IL-1*β*, IL-12, TNF-*α*, and IL-6, without reduction or with increase in production of anti-inflammatory cytokines such as IL-10, by macrophages, and by monocytes undergoing reprogramming [7, 8]. These mechanisms augment the immunosuppression already present in the patient with cancer. Inflammatory signs and symptoms are usually described as attenuated in infections of febrile neutropenic patients [9], assuming that inflammatory response is also attenuated, but which is in fact understudied. Although mediators such as IL-8 and IL-6 have been evaluated in febrile neutropenic children as risk markers for severe infections, the kinetics of cytokines during the time of fever or occurrence of sepsis has been rarely studied [10]. We found no data, for example, about the behavior of the IL-23/IL-17 axis, associated with protective immunity at mucosal surfaces [11], a decisive factor in bacterial translocation in patients undergoing chemotherapy. In addition, there are no data on potential pro- or anti-inflammatory effects of filgrastim, an analogue of cytokine granulocyte colony-stimulating factor (G-CSF) widely used in the treatment of neutropenia.

The main objectives of this study were to investigate the dynamics of the inflammatory and anti-inflammatory responses in febrile neutropenic children, throughout the progression of intercurrent sepsis and recovery of leukocytes, by means of the measurement of circulating cytokines (IL-1*β*, TNF-*α*, IL-10, IL-12/23p40, IL-21, IL-6, IL-8, IL-17, G-CSF, and GM-CSF) in plasma. Also, we aimed to evaluate the performance of these cytokines as sepsis discriminators compared with procalcitonin (PCT) and a clinical/laboratory model for risk assessment on the first day of fever. Secondary objectives were to assess the possible influence of filgrastim on the expression of these markers and the proper functioning of IL-17/IL-23 axis in these patients.

## 2. Methods

The protocol was approved by the local Ethics Committee. After informed consent was obtained from parents, patients aged up to 18 years (incomplete) were sequentially included, according to the following criteria: presence of an oncologic disease and low- or high-risk for febrile neutropenia (according to a high-risk assessment model based on clinical data and CRP; Table 1). Patients were followed for 60 days. Sepsis was defined according to the 2005 consensus [12]. Blood samples (2 mL) were collected from patients on the first day of fever in children with neutropenia and then on days 2, 3, 5, and 14. After centrifugation and separation of plasma, samples were frozen initially at −20°C and posteriorly at −80°C. IL-1*β*, TNF-*α*, IL-10, IL-12/23p40, IL-21, IL-6, IL-8, IL-17, G-CSF, and GM-CSF were measured using the Cytometric Bead Array (CBA) Flex Set from Becton & Dickinson (BD Biosciences, San Jose, CA, USA). PCT was measured by ELISA (Human Procalcitonin ELISA, Biovendor, Brno, Czech Republic). Additional blood samples (2 mL) were collected in EDTA tubes for blood cell analyses, which were performed in automated counter (Cell-Dyn Ruby, Abbott, Illinois, USA), with microscopic examination of blood smear by the hematologist when necessary.

Data were analyzed with SPSS 20.0 software (IBM Corp., Armonk, NY, USA) and Minitab 17 (Minitab Inc., State College, PA, USA). The variables were described as medians and interquartile ranges. The value of significance was *P* < 0.05. Differences between groups were evaluated using the Mann–Whitney *U* test. Correlations were assessed by the Spearman test and linear regression. Bootstrap was used to determine the confidence intervals and the standard error with resampling of at least 1000 tables. ROC curves were used to assess usefulness of markers as discriminators of sepsis. Optimal cutoff points were determined by the Youden *J* index (*J* = {maximum sensitivity + specificity − 1}) [13]. Likelihood ratios were calculated to assess the possible influence of the tests in clinical decisions [14].

## 3. Results

We included 35 patients, whose epidemiological characteristics are shown in Table 2. Circulating cytokine, PCT, and CRP levels at admission and during follow-up are shown in Table 3.

**Figure 1:**
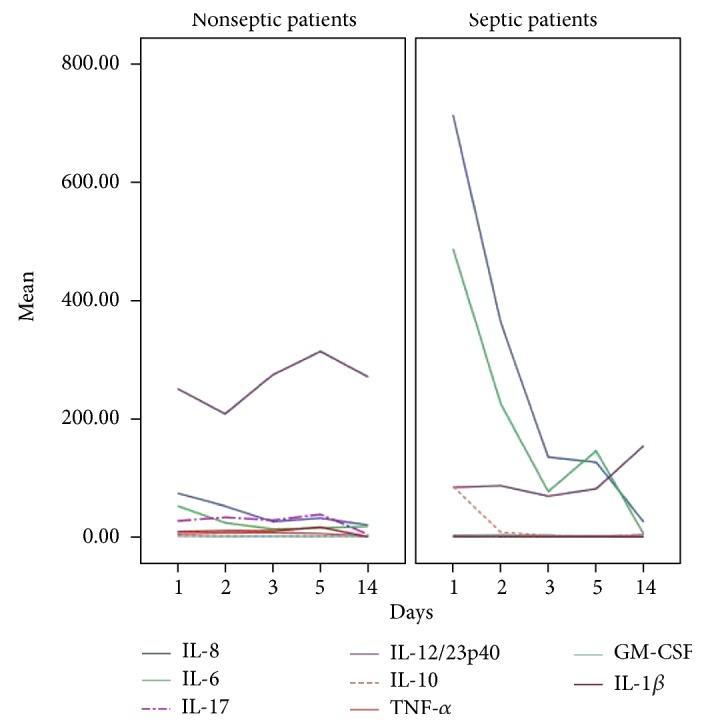
Kinetics of cytokines (observed means in pg/mL) on days 1, 2, 3, 5, and 14 of fever in febrile neutropenic patients. For better visualization, IL-21 (near absent expression) and G-CSF (very high levels due to filgrastim administration) were not shown.

Thirteen patients (37.1%) developed sepsis within 72 hours after diagnosis of febrile neutropenia. Of these, six showed clinical signs of severe sepsis and four had septic shock. Nine of the thirteen patients who developed sepsis were initially categorized as high risk. Bacteria were found in ten septic patients: in eight patients via blood culture (four catheter-drawn and four venipuncture) and in two patients via urine cultures. Blood cultures were positive for three coagulase-negative staphylococci (two *Staphylococcus epidermidis*, one *Staphylococcus caprae*), one *Enterococcus raffinosus*, two *E. coli*, and one *Enterobacter* sp. In one case, the same bacterium (*Pseudomonas oryzihabitans*) was isolated both in peripheral and catheter blood cultures. Two urine cultures were positive *for Klebsiella pneumoniae*. In three patients with signs of shock, no bacteria were recovered. There were no deaths within 60 days after enrollment. Six patients (17.1%) were admitted to the intensive care unit (ICU). The kinetics of cytokines in septic and nonseptic patients is shown in Figure 1.

### 3.1. Factor Analysis

Cytokines were included in a factor analysis model comprising the five moments of measurement. The cumulative total variance explained by the model was 94.1%, with a Kaiser–Meyer–Olkin measure of sampling adequacy of 0.85 and Bartlett's sphericity test with *P* < 0.0001. The model reduced the 10 variables to three factors, whose loadings are shown in Table 4. G-CSF presented communality <0.4 and was removed.

The scores computed for factors 1, 2, and 3 were analyzed by mixed models with repeated measurements and sepsis as fixed effects. Factor 2, representing IL-6, IL-8, and IL-10 loadings, showed different estimated means between sepsis and nonsepsis (−0.34 and 0.33; *P* = 0.000) and negatively correlated (Spearman) with number of neutrophils (coefficient: −0.56; *P* = 0.000), lymphocytes (−0.36; *P* = 0.001), and monocytes (−0.43; *P* = 0.000). Factor 1, representing IL-1*β*, IL-17, IL-12/23p40, IL-17, IL-21, and TNF-*α* loadings, showed a negative correlation (coefficient: −0.39; *P* = 0.013) with CRP and positive with the number of neutrophils (coefficient: 0.36; *P* = 0.001) and monocytes (coefficient: 0.23; *P* = 0.035). Factor 3, comprising GM-CSF loading, showed a negative correlation with the total number of monocytes (coefficient: −0.23; *P* = 0.037). Factors 1 and 3 showed no differences in estimated means for sepsis.

### 3.2. Mixed Models for Individual Markers

IL-6 showed significant temporal variation (negative slope) between day 1 and day 3 (estimated mean difference: −209 pg/mL; *P* = 0.000), between day 1 and day 5 (mean difference: −173.7; *P* = 0, 02), and between day 1 and day 14 (−242.5; *P* = 0.000). In the evaluation of sepsis as a fixed effect, the estimated means were 181.1 pg/mL for septic patients and 24.8 for the others (*P* = 0.000).

IL-8 showed different estimated means for patients who developed sepsis (265.9 pg/mL) and patients who did not (40.9; *P* = 0.0000). It also showed significant temporal variation with negative slope between day 1 and day 3 (estimated mean difference: −364 pg/mL; *P* = 0.002), between day 1 and day 5 (−363.5 pg/mL; *P* = 0.003), and between day 1 and day 14 (−434.7; *P* = 0.000).

**Figure 2:**
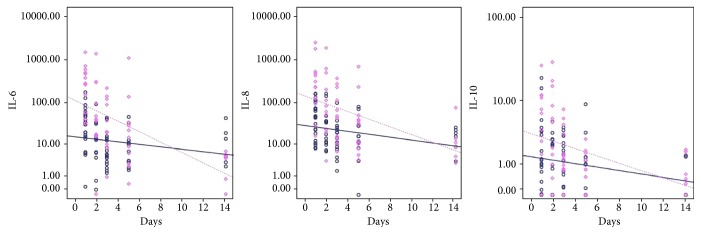
Fitting lines of IL-6, IL-8, and IL-10 (base-10 log scale) for the occurrence of sepsis (dashed line and diamonds) or not (solid line and circles) on days 1, 2, 3, 5, and 14 of the onset of fever.

For IL-10, the estimated means were also different between patients with sepsis and nonseptic patients (8.5 pg/mL and 1.9; *P* = 0.004). There was significant temporal variation, with negative slope between day 1 and day 2 (estimated mean difference: −11.5; *P* = 0.038), between day 1 and day 3 (−14; *P* = 0.011), between day 1 and day 5 (difference: −14.9; *P* = 0.007), and between day 1 and day 14 (−15.6; *P* = 0.005).  Figure 2 shows the fitting lines for IL-6, IL-8, and IL-10, for septic and nonseptic patients.

IL-12/23p40 had higher estimated means for the nonoccurrence of sepsis (263.3 pg/mL versus 93.5 for sepsis; *P* = 0.044). There was no significant temporal variation.

**Figure 3:**
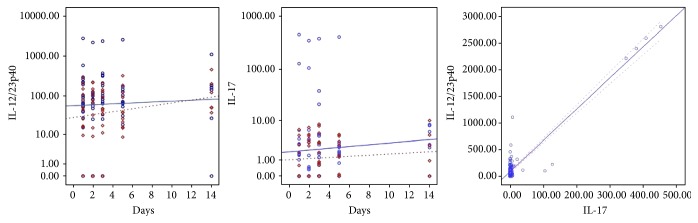
Fitting lines for sepsis (dashed line and diamonds) or not (solid line and circles) for IL-12/23p40 (right) and IL-17 (center), in base-10 log scale. On the left, the linear regression between values of IL-12/23p40 and IL-17 (*R*^2^ = 0.88).

IL-17 also showed no significant temporal variation. For patients who developed sepsis, the estimated means were 2.22 pg/mL, and for the others, 26.5 pg/mL (*P* = 0.04). The correlation between IL-17 and IL-12/23p40 was linear with *R*^2^ = 0.88 (*P* = 0.000).  Figure 3 shows the fitting lines of IL-12/23p40 and IL-17 along the measurements and their linear regression.

There was no significant temporal variation of IL-1*β*. For patients with sepsis, the estimated means were 7.7, and for nonseptic patients, 0.41 pg/mL (*P* = 0.11). IL-21 showed expression in only one nonseptic patient on day 1, with erratic expression in a few other patients along the days, and was not analyzed in mixed models.

GM-CSF showed estimated means of 1.3 and 1.8 pg/mL for patients with sepsis or not (*P* = 0.15). There was no significant temporal variation.

TNF-*α* had estimated means of 1.44 pg/mL for patients with sepsis, and 4.6 (*P* = 0.16) for nonseptic patients, also with no significant temporal variation.

**Figure 4:**
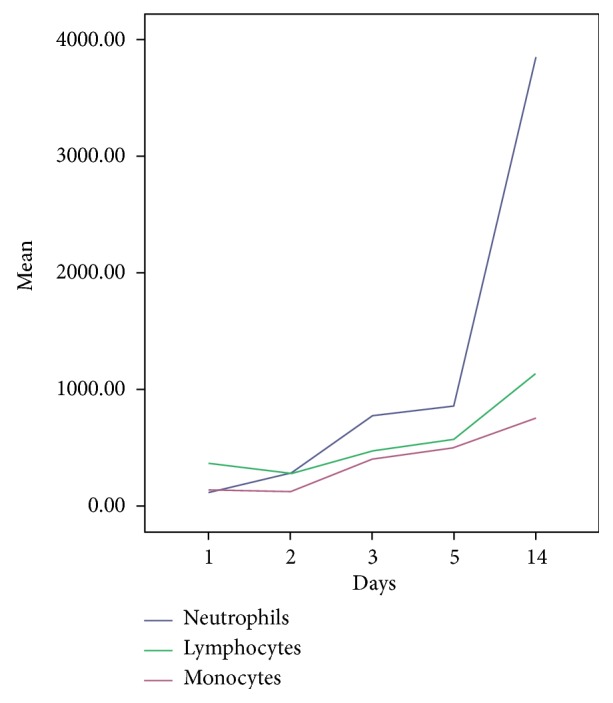
Temporal evolution of leukocytes along the days (in means, cells/*μ*L).

Leukocytes (total counts) showed significant temporal variation (positive slope), with a mean difference of +1369 cells/*μ*L between day 1 and day 5 (*P* = 0.003) and +3059 cells/*μ*L between day 1 and day 14. For septic patients, the mean estimated leukocyte counts were 1608 cells/*μ*L versus 2067 cells/*μ*L for nonseptic patients (*P* = 0.3). For neutrophils, the estimated means were 967 and 745 cells/*μ*L for septic patients or nonseptic patients (*P* = 0.46). There was an increase along the days (mean difference: +1850 cells/*μ*L to day 14; *P* = 0.036). Lymphocytes also showed an increase up to day 14 (+745 cells/*μ*L; *P* = 0.02). For septic patients or nonseptic patients, the estimated means were 483.1 and 292.2 cells/*μ*L (*P* = 0.08).  Figure 4 shows the temporal variation in the number of leukocytes.

Monocytes showed significant temporal variation between day 1 and day 3 (positive slope, mean difference: 235 cells/*μ*L; *P* = 0.03), between day 1 and day 5 (+371.8 cells/*μ*L; *P* = 0.03), and between day 1 and day 14 (+592 cells/*μ*L; *P* = 0.001). For septic patients or nonseptic patients, the estimated means were 347 and 391 cells/*μ*L (*P* = 0.63). When the occurrence of positive blood cultures was added to the model, the estimated means were 107.8 and 257.8 cells/*μ*L for septic or nonseptic patients (*P* = 0.017).

Platelets decreased between the days 1 and 2 (mean difference: −46,827 platelets/*μ*L; *P* = 0.027), increasing until day 14 (+48,361 platelets/*μ*L; *P* = 0.028). There were no significant differences related to the occurrence of sepsis.

The CRP showed estimated means of 106.1 mg/L for septic and 52.9 for nonseptic patients (*P* = 0.14), with significant temporal variation from days 1 to 5 (negative slope, mean difference: −141.5).

### 3.3. Filgrastim and Cytokine Levels

Among the patients included, 19 were receiving filgrastim at the time of inclusion in accordance with chemotherapy protocols. Eight (42%) of these patients developed sepsis. On the first day of fever, IL-6, IL-8, IL-10, and G-CSF showed differences in the distribution or in the medians of patients who received filgrastim and those who did not. For IL-6, IL-8, and G-CSF, the differences on the Mann–Whitney *U* test remained significant after removal of septic patients: for IL-8, *P* = 0.042, 99% CI 0.037–0.047; IL-6, *P* = 0.006, 99% CI 000.3–0.006; and G-CSF, *P* = 0.000. Despite the higher median of PCT in the patients who received filgrastim, the difference was not significant (Table 5).

In the mixed model, G-CSF presented different estimated means between septic and nonseptic patients (2685.76 and 1509.6; *P* = 0.009). The temporal variation was significant (negative slope), with a mean difference of −1608 between day 1 and day 5 and −3191 between day 1 and day 14.

### 3.4. Analysis of the Biomarkers as Sepsis Discriminators

Septic and nonseptic patients showed significant differences at day 1 by the Spearman test for IL-8, IL-6, IL-10, and PCT. These markers were tested with ROC curves as discriminators of sepsis, and their performances are shown in Table 6. The performances of the cutoff values of the biomarkers, their combinations, and the comparison with the high-risk assessment model are shown in Table 7.

Optimal cutoff points were determined by the Youden *J* index, ranging from 0 (zero—no accuracy) and 1 (perfect test) [13]. For LR+, values >10 have a great impact on diagnosis; between 5 and 10, moderate impact; and between 2 and 5, low impact. For LR−, values <0.1 have a great impact. The impact is moderate for values between 0.1 and 0.2 and low for values between 0.2 and 0.5. For both, values equal to 1 show no impact [14].

The use of filgrastim caused minor changes in the areas under the ROC curves of IL-8 (0.88; *P* = 0.04) and IL-6 (0.78; *P* = 0.037) for sepsis discrimination. For patients who did not receive filgrastim, the AUC was 0.83 for IL-8 (*P* = 0.034) and 0.9 for IL-6 (*P* = 0.009).

IL-8 and PCT correlated with the duration of fever by linear regression (IL-8: *P* < 0.0001, *R*^2^ = 0.42; PCT: *P* < 0.0001, *R*^2^ = 0.45). IL-6 also showed a correlation with the time of fever, but only on Spearman's test (*P* = 0.001).

## 4. Discussion

In our septic patients, the cytokines with a greater expression were IL-6 and IL-8, which are produced by multiple types of cells, and were apparently spared by chemotherapy. IL-6 is produced by lymphocytes, macrophages, dendritic cells, endothelial cells, fibroblasts, and smooth muscle cells in response to stimulation with LPS, IL-1, and TNF-*α*. IL-6 levels tend to remain high in sepsis, and one of its biological effects is the induction of fever [15, 16]. IL-8 also is produced by fibroblasts and epithelial, mesothelial, and endothelial cells, in addition to monocytes and neutrophils [17]. TNF-*α* and IL-1*β*, produced primarily by activated macrophages, had little expression. The peaks of systemic release of TNF-*α* and IL-1*β* occur quickly (90 min after endotoxin administration in human volunteers for TNF and 180 min for IL-1*β*) [18]. In addition, both do not show consistent patterns of gene expression and are highly variable between individuals, and their expression is downregulated by IL-6 and IL-10 [16]. The inflammatory response in sepsis of febrile neutropenia appears to be similar to that in the nonneutropenic patients [19]. In our study, the anti-inflammatory response, characterized by IL-10 expression, accompanied IL-8 and IL-6 variance in time, showing that pro- and anti-inflammatory responses occur early and simultaneously. The shared variance of IL-8, IL-6, and IL-10 (factor 2 in factor analysis) was correlated with the development of sepsis. The shared variance of IL-21, IL-17, IL-12/23p40, TNF-*α*, and IL-1*β* (factor 1) correlated positively with neutrophil and lymphocyte counts and negatively with CRP, suggesting that this variance may be related to homeostasis regarding sterile inflammation, because CRP means decreased toward day 5, as leukocytes recovered.

The kinetics of cytokines observed in the mixed models showed that there was initially a “storm” of IL-6 and IL-8, which was sustained on the first 2 days, correlating with the time of fever and decreasing on the third day, in a proinflammatory response similar to what is described in nonneutropenic patients [20]. The concomitant anti-inflammatory response of IL-10 also decreases sharply after the second day. The fitting lines show that the expression of these cytokines becomes similar between nonseptic and septic patients at approximately day 10.

IL-12 and IL-23 share the subunit p40, and it was this subunit that was measured in this study. Therefore, it is not possible to determine the individual contribution of IL-12 or IL-23. However, the linear relationship between the measurements of IL-12/23p40 and IL-17 (*R*^2^ = 0.88) suggests that we have been dealing almost exclusively with IL-23, which is the inducer of IL-17, and that observed fluctuations must belong to axis IL-23/Th17. IL-12 and IL-23 are secreted mainly by resident tissue macrophages and dendritic cells. Whereas IL-12 promotes differentiation of naïve CD4 cells in Th1 producing interferon (IFN-*γ*) cells, IL-23 does not promote Th differentiation directly due to the absence or low expression of IL-23 receptors (IL-23R) in naïve lymphocytes. In mice, the transforming growth factor beta (TGF-*β*), IL-1, and IL-6 induce the expression of retinoic acid-related orphan receptor gamma (ROR-*γ*t), a lineage-specific transcription factor that induces the transcription of IL-17A and IL-17F, encoding genes in CD4+ naïve cells [21, 22]. The later stages of Th17 differentiation (clonal expansion, phenotypic stabilization with IL-17 production) apparently depend on IL-23 expression [23]. Although the IL-23/Th17 axis has been described as the orchestrator of the recruitment and activation of neutrophils and a key player in chronic inflammation and autoimmunity, recent evidence suggests that the effector cytokines Th17 (IL-17 and IL-22) play a crucial role in maintaining mucosal immunity, protecting against extracellular bacteria and fungi. This includes the intestinal lining, where these cytokines assist in maintaining the physical barrier intact and induce the expression of antimicrobial peptides to prevent invasion by luminal bacteria [24]. It is well known that the intestinal flora is a major source of serious infections in patients undergoing chemotherapy [25]. Our nonseptic patients had higher IL-12/23p40 and IL-17 means, suggesting integrity of the IL-23/Th17 axis, which is not apparent in the septic patients. Five of the eight isolates from blood cultures in our septic patients were intestinal bacteria. The low expression of IL-17 can be explained by depletion and impairment of naïve Th cells after cytotoxic chemotherapy and by the reduction in absolute numbers of lymphocytes producing IL-17 in septic patients [26, 27]. Depletion of CD4+ cells may also explain the almost-absent expression of IL-21, another cytokine involved in Th17 differentiation: IL-6 induces IL-21 expression, which in turn amplifies an autocrine loop to induce more IL-21 and IL-23 receptors in naïve CD4+ cells. Both IL-21 and IL-23 together with TGF-*β* can induce IL-17 expression irrespective of IL-6 [28]. This deficiency in the IL-23/Th17 axis may be an important immunosuppressant factor in febrile neutropenic sepsis. It is not possible, however, to establish causal relationships.

Various authors have studied the role of biomarkers for diagnosing bacterial infections in children with febrile neutropenia. Stryjewski et al. evaluated IL-6, IL-8, and PCT for discrimination of bacterial sepsis in neutropenic children and observed an excellent discriminating power of PCT 24 hours after admission. For IL-6 and IL-8, the power of discrimination was considered good after 24 and 48 hours after admission, and the best observed result was the combination of IL-8 and PCT (sensitivity 94% and specificity 90% for PCT >500 and IL-8 >20 pg/mL) [29]. Urbonas et al. described an IL-10 sensitivity of 73% and a specificity of 92% (cut-off: 18 pg/mL) for discrimination of sepsis or bacteremia in febrile neutropenic children [30]. In our study, we tried to evaluate the usefulness of other cytokines as discriminators of sepsis on admission, within the first 24 hours of fever, when clinical signs of severe infections can still be subtle or absent in neutropenic children. We confirmed the observations of other authors about the good performance of IL-6, IL-8, IL-10, and PCT. We also demonstrated that the combination of one of these markers improves the performance of a risk assessment model (e.g., IL-8 >240 + high risk, or PCT >180 + high risk). In this sense, IL-8 has been validated by Miedema et al., being useful when combined with clinical parameters to stratify patients at risk for bacterial infections, in order to shorten antibiotic treatment in selected patients [31]. The other cytokines in our study (IL-1*β*, TNF-*α*, IL-12/23p40, IL-21, IL-17, G-CSF, and GM-CSF) do not appear to be useful markers for clinical decisions regarding sepsis.

High local and systemic concentrations of G-CSF are found in patients with infections. At sites of infection, G-CSF is produced by monocytes and macrophages [32]. In animal models of sepsis, pretreatment with G-CSF attenuated serum levels of TNF induced by LPS, as well as ex vivo TNF release in different populations of macrophages. This did not occur with the addition of G-CSF in cultures of macrophages, indicating that pretreatment alters the cytokine response in sepsis indirectly, by indefinite factors or effector cells [33]. In blood samples from volunteers treated with G-CSF subcutaneously 1 day before collection, ex vivo stimulation with LPS caused an increase in IL-6, IL-8, IL-10, G-CSF, IL-1 receptor antagonist (IL-1ra), and soluble TNF receptor release and reduced the release of IFN-*γ* and GM-CSF [34]. These studies show inflammatory and anti-inflammatory effects of G-CSF. We found no studies about effects of filgrastim on the inflammatory response in febrile neutropenic children. In our study, filgrastim administration apparently upregulates the systemic release of IL-8, IL-6, and IL-10 on the first day of fever, with little impact on sepsis discrimination capability.

This study has several limitations, the most obvious being the small number of patients. The number of biomarkers that we measured was also limited, and we left out important cytokines in the inflammatory and anti-inflammatory response, such as IFN-*γ* and IL-4. Another limitation was the commercial nonavailability of IL-23p19 for the CBA method at the time. However, as strengths, we raised the need to clarify in future studies the role of filgrastim in inflammatory and anti-inflammatory responses in febrile neutropenic children and also to elucidate the role of IL-23-Th17 axis in the sepsis of these patients.

## 5. Conclusions

During febrile neutropenia, pro- and anti-inflammatory cytokines are detected simultaneously in the blood in children with oncological diseases. We observed deficiencies in the IL-23-Th17 axis and the IL-21 expression in these patients, which may characterize aspects of immunosuppression in those progressing to sepsis. The study further supports the usefulness of IL-8, IL-6, IL-10, and PCT as biomarkers of sepsis in febrile neutropenic children. Filgrastim appears to regulate upward expression of these markers on the first day of fever, with little impact on diagnostic performance.

## Figures and Tables

**Table 1:** Definition of febrile neutropenia and risk assessment model for serious infections.

*Definition of low-risk febrile neutropenia*
(i) Neutrophil count (<500 cells/*μ*L)
(ii) Fever (one axillary temperature >38°C, or 3 measures >37.5 and <38°C, with a 4-hour interval between measurements, in 24 hours)
(iii) Solid tumors without comorbidities (see below)
(iv) Acute lymphoblastic leukemia (ALL) in remission without comorbidities
(v) Lymphomas in remission without comorbidities (vi) C-reactive protein (CRP) (<90 mg/L)
(vii) Treatment: outpatient (daily monitoring until the end of antibiotic therapy)
*Definition of high-risk febrile neutropenia*
(i) Neutrophil count (<500 cells/*μ*L)
(ii) Fever (one axillary temperature >38°C, or 3 measures >37.5 and <38°C, with a 4-hour interval between measurements, in 24 hours)
(iii) Acute myeloid leukemia (AML) in activity or remission
(iv) Solid tumors with comorbidities (see below) or treated with high-dose chemotherapy
(v) Acute lymphoblastic leukemia in activity or comorbidities
(vi) Lymphomas in activity or comorbidities
(vii) CRP >90 mg/L
(viii) Treatment: in hospital
*Comorbidities*
(i) Meningitis, pneumonia, diarrhea or vomiting, mucositis grade 3 or 4, hypotension, signs of sepsis or bacteremia, metabolic changes, disease activity with medullary invasion, age <3 years, suspected catheter-related infections, severe abdominal pain or abdominal distension, radiologic findings suggestive of typhlitis, perianal abscess

**Table 2:** General data (*N*, %). IQR: interquartile range.

*N*	35
Age (years, median, IQR)	5.84 (3.7–10.3)
Male	10 (28.6%)
Received antibiotics	35 (100%)
Diagnoses	
Acute lymphocytic leukemia	6 (17.1%)
Acute myelogenous leukemia	3 (8.6%)
Non-Hodgkin's lymphoma	6 (17.1%)
Wilms' tumor	4 (11.4%)
Primitive neuroectodermal tumor (PNET)	2 (5.7%)
Retinoblastoma	2 (5.7%)
Ewing's sarcoma	2 (5.7%)
Chronic myeloid leukemia	1 (2.9%)
Other solid tumors	9 (25.7%)
Received filgrastim	19 (54.2%)
Days receiving filgrastim before day 1 of the study (mean, range)	4.5 (1–7)
Low risk	11 (31.5%)
High risk (hospitalized)	24 (68.5%)

**Table 3:** Values of biomarkers in the 5 days of measurements in median and interquartile ranges (25–75 percentiles).

Day	Median	IQR p25–75	Median	IQR p25–75
	IL-8 (pg/mL)		IL-12/23p40 (pg/mL)	
1	89.9	32.8–243.2	63.7	30.6–203.8
2	46.2	19.2–133.4	67.3	10.4–110.9
3	24.9	13.5–58.6	77.2	34.5–162.8
5	20.5	13.5–50.7	66.2	26.3–128.5
14	16.3	8.9–24.6	132.6	51–191.9
	IL-6 (pg/mL)		IL-10 (pg/mL)	
1	57.3	31–198.2	2.2	0.6–10.2
2	21.6	10.1–49.9	2.6	0.1–5.4
3	12.7	5.9–36	1.8	0.8–3.3
5	11.9	4.7–35.6	1.1	0–1.7
14	6.3	3.8–7.7	0.4	0–1.7
	IL-21 (pg/mL)		TNF-*α* (pg/mL)	
1	0	0-0	0	0–2.65
2	0	0-0	0	0–1.5
3	0	0-0	0.6	0–2.5
5	0	0-0	0.3	0–1.7
14	0	0-0	1.7	0–2.8
	IL-1*β* (pg/mL)		G-CSF (pg/mL)	
1	0	0-0	2182.7	282.7–5739.5
2	0	0	2350.2	356–4793.9
3	0	0	947.9	139.9–4125
5	0	0-0.5	177	31–2232.4
14	0	0	28.1	15.5–50
	IL-17 (pg/mL)		GM-CSF (pg/mL)	
1	0	0–2.6	0.1	0–2.8
2	0.5	0–3.7	0.9	0–3.4
3	1.1	0–4.9	0.6	0–2.4
5	1.6	0–2.8	0.6	0–1.9
14	2.8	0.5–6.4	1.6	0.4–2
	C-reactive protein (mg/L)		Procalcitonin (pg/mL)	
1	56	28.9–98	149.1	0–909.6
2	78.5	61.8–95.3		
3	87.2	54.2–130.6		
5	47.7	16.2–87.5		
14	7.7	5.7–7.2		

**Table 4:** Factor analysis loadings of the three factors of the solution. Loadings > 0.7 (in bold) confirm that variables are represented by each factor.

	1	2	3
IL-8	−0.04	**0.95**	0.19
IL-6	−0.04	**0.91**	0.23
IL-21	**0.99**	−0.02	0.02
IL-17	**0.98**	−0.03	0.00
IL-12/23p40	**0.96**	−0.06	−0.02
IL-10	0.01	**0.89**	−0.24
TNF-*α*	**0.99**	0.00	0.02
GM-CSF	0.03	0.11	**0.98**
IL-1*β*	**0.99**	−0.02	0.02

**Table 5:** Medians of biomarkers (pg/mL) on the day 1 of fever in patients receiving filgrastim or not. ^∗^Mann–Whitney and median tests; ^‡^Mann–Whitney test; ^†^determined by Monte Carlo simulation with 10,000 tables.

	Filgrastim	No filgrastim	*P*	*P* 99% CI^†^
G-CSF	5838	302.9	0.000^∗^	0.000-0.000
IL-8	155.4	58.2	0.011^‡^	0.006–0.011
IL-6	132	39.5	0.007^∗^	0.003–0.007
IL-10	5.51	1.2	0.004^‡^	0.002–0.04
PCT	223.3	98.1	0.07^‡^	0.06–0.08

**Table 6:** Performance of IL-6, IL-8, PCT, and IL-10 for discrimination of sepsis on day 1 of fever. AUC: area under the ROC curve.

	AUC	Standard error	*P*	95% CI (sample)	95% CI (Bootstrap)	Youden (*J*)
IL-6	0.87	0.062	0.000	0.75–0.99	0.76–0.8	0.65
IL-8	0.86	0.074	0.000	0.72–1	0.83–0.87	0.7
PCT	0.89	0.054	0.000	0.78–0.99	0.87–0.90	0.64
IL-10	0.83	0.08	0.001	0.67–0.98	0.79–0.83	0.6

**Table 7:** Performance of cutoff values and combinations of the biomarkers IL-6, IL-8, IL-10, and PCT (all in pg/mL) as sepsis discriminators on day 1 of fever, compared with the high-risk assessment model. *S* = sensitivity; *E* = specificity; PPV = positive predictive value; NPV = negative predictive value; LR+ = positive likelihood ratio; LR− = negative likelihood ratio.

	IL-6 > 170	IL-8 > 240	PCT > 180	IL-10 > 6	High risk
*S*	0.69	0.69	0.80	0.69	1.00
*E*	0.95	1.00	0.68	0.86	0.44
PPV	0.90	1.00	0.50	0.75	0.42
NPV	0.84	0.85	0.89	0.83	1.00
LR+	9.00	−	1.00	3.00	0.71
LR−	0.19	0.18	0.12	0.21	0.00
Youden *J*	0.65	0.69	0.48	0.56	0.44

	IL-8 > 240 + high risk	PCT > 180 + high risk	IL-10 > 6 + PCT > 180	IL-8 > 100 + PCT > 100	IL-6 > 50 + PCT > 100

*S*	0.69	0.77	0.69	0.85	0.85
*E*	1.00	0.86	0.91	0.91	0.82
PPV	1.00	0.77	0.82	0.85	0.73
NPV	0.85	0.86	0.83	0.91	0.90
LR+	—	3.33	4.50	5.50	2.75
LR−	0.18	0.16	0.20	0.10	0.11
Youden *J*	0.69	0.63	0.60	0.76	0.66

## Abbreviations

CBA: Cytometric bead array
CRP: C-reactive protein
DAMP: Damage associated molecular patterns
ELISA: Enzyme-linked immunosorbent assay
G-CSF: Granulocyte colony-stimulating factor
GM-CSF: Granulocyte-macrophage colony-stimulating factor
ICU: Intensive care unit
IL: Interleukin
PAMP: Pathogen-associated molecular patterns
PCT: Procalcitonin
ROC curve: Receiver operating characteristic curve
SIRS: Systemic inflammatory response syndrome
Th: T helper cells
TNF: Tumor necrosis factor.

## References

[1] Salomao R., Brunialti M. K., Rapozo M. M., Baggio-Zappia G. L., Galanos C., Freudenberg M. (2012). Bacterial sensing, cell signaling, and modulation of the immune response during sepsis. *Shock*.

[2] Wennerås C., Hagberg L., Andersson R. (2014). Distinct inflammatory mediator patterns characterize infectious and sterile systemic inflammation in febrile neutropenic hematology patients. *PloS One*.

[3] Kono H., Rock K. L. (2008). How dying cells alert the immune system to danger. *Nature Reviews. Immunology*.

[4] Robertson C. M., Coopersmith C. M. (2006). The systemic inflammatory response syndrome. *Microbes and Infection*.

[5] Bodey G. P. (2009). The changing face of febrile neutropenia-from monotherapy to moulds to mucositis. Fever and neutropenia: the early years. *The Journal of Antimicrobial Chemotherapy*.

[6] Osuchowski M. F., Craciun F., Weixelbaumer K. M., Duffy E. R., Remick D. G. (2012). Sepsis chronically in MARS: systemic cytokine responses are always mixed regardless of the outcome, magnitude, or phase of sepsis. *Journal of Immunology*.

[7] Santos S. S., Carmo A. M., Brunialti M. K. (2016). Modulation of monocytes in septic patients: preserved phagocytic activity, increased ROS and NO generation, and decreased production of inflammatory cytokines. *Intensive Care Medicine Experimental*.

[8] Biswas S. K., Lopez-Collazo E. (2009). Endotoxin tolerance: new mechanisms, molecules and clinical significance. *Trends in Immunology*.

[9] Freifeld A. G., Bow E. J., Sepkowitz K. A. (2011). Clinical practice guideline for the use of antimicrobial agents in neutropenic patients with cancer: 2010 update by the Infectious Diseases Society of America. *Clinical Infectious Diseases*.

[10] Engel A., Kern W. V., Mürdter G., Kern P. (1994). Kinetics and correlation with body temperature of circulating interleukin-6, interleukin-8, tumor necrosis factor alpha and interleukin-1 beta in patients with fever and neutropenia. *Infection*.

[11] Maloy K. J., Kullberg M. C. (2008). IL-23 and Th17 cytokines in intestinal homeostasis. *Mucosal Immunology*.

[12] Goldstein B., Giroir B., Randolph A. (2005). International pediatric sepsis consensus conference: definitions for sepsis and organ dysfunction in pediatrics. *Pediatric Critical Care Medicine*.

[13] Ruopp M. D., Perkins N. J., Whitcomb B. W., Schisterman E. F. (2008). Youden index and optimal cut-point estimated from observations affected by a lower limit of detection. *Biometrical Journal*.

[14] Hayden S. R., Brown M. D. (1999). Likelihood ratio: a powerful tool for incorporating the results of a diagnostic test into clinical decision making. *Annals of Emergency Medicine*.

[15] Kuhns D. B., Alvord W. G., Gallin J. I. (1995). Increased circulating cytokines, cytokine antagonists, and E-selectin after intravenous administration of endotoxin in humans. *Journal of Infectious Diseases*.

[16] Schulte W., Bernhagen J., Bucala R. (2013). Cytokines in sepsis: potent immunoregulators and potential therapeutic targets—an updated view. *Mediators of Inflammation*.

[17] Matsushima K., Oppenheim J. J. (1989). Interleukin 8 and MCAF: novel inflammatory cytokines inducible by IL 1 and TNF. *Cytokine*.

[18] Cannon J. G., Tompkins R. G., Gelfand J. A. (1990). Circulating interleukin-1 and tumor necrosis factor in septic shock and experimental endotoxin fever. *The Journal of Infectious Diseases*.

[19] Regazzoni C. J., Khoury M., Irrazabal C. (2003). Neutropenia and the development of the systemic inflammatory response syndrome. *Intensive Care Medicine*.

[20] Hotchkiss R. S., Monneret G., Payen D. (2013). Immunosuppression in sepsis: a novel understanding of the disorder and a new therapeutic approach. *The Lancet Infectious Diseases*.

[21] Ivanov I. I., McKenzie B. S., Zhou L. (2006). The orphan nuclear receptor RORγt directs the differentiation program of proinflammatory IL-17+ T helper cells. *Cell*.

[22] Teng M. W., Bowman E. P., McElwee J. J. (2015). IL-12 and IL-23 cytokines: from discovery to targeted therapies for immune-mediated inflammatory diseases. *Nature Medicine*.

[23] Bosmann M., Ward P. A. (2012). Therapeutic potential of targeting IL-17 and IL-23 in sepsis. *Clinical and Translational Medicine*.

[24] Rendon J. L., Choudhry M. A. (2012). Th17 cells: critical mediators of host responses to burn injury and sepsis. *Journal of Leukocyte Biology*.

[25] Peterson D. E., Cariello A. (2004). Mucosal damage: a major risk factor for severe complications after cytotoxic therapy. *Seminars in Oncology*.

[26] Heitger A., Winklehner P., Obexer P. (2002). Defective T-helper cell function after T-cell-depleting therapy affecting naïve and memory populations. *Blood*.

[27] Brunialti M. K., Santos M. C., Rigato O., Machado F. R., Silva E., Salomao R. (2012). Increased percentages of T helper cells producing IL-17 and monocytes expressing markers of alternative activation in patients with sepsis. *PloS One*.

[28] Zhou L., Ivanov I. I., Spolski R. (2007). IL-6 programs T(H)-17 cell differentiation by promoting sequential engagement of the IL-21 and IL-23 pathways. *Nature Immunology*.

[29] Stryjewski G. R., Nylen E. S., Bell M. J. (2005). Interleukin-6, interleukin-8, and a rapid and sensitive assay for calcitonin precursors for the determination of bacterial sepsis in febrile neutropenic children. *Pediatric Critical Care Medicine*.

[30] Urbonas V., Eidukaitė A., Tamulienė I. (2012). Increased interleukin-10 levels correlate with bacteremia and sepsis in febrile neutropenia pediatric oncology patients. *Cytokine*.

[31] Miedema K. G., Tissing W. J., Abbink F. C. (2016). Risk-adapted approach for fever and neutropenia in paediatric cancer patients—a national multicentre study. *European Journal of Cancer*.

[32] Quinton L. J., Nelson S., Boe D. M. (2002). The granulocyte colony-stimulating factor response after intrapulmonary and systemic bacterial challenges. *The Journal of Infectious Diseases*.

[33] Gorgen H. T., Leist M., Niehorster M. (1992). Granulocyte colony-stimulating factor treatment protects rodents against lipopolysaccharide-induced toxicity via suppression of systemic tumor necrosis factor-a. *Journal of Immunology*.

[34] Hartung T., Döcke W. D., Gantner F. (1995). Effect of granulocyte colony-stimulating factor treatment on ex vivo blood cytokine response in human volunteers. *Blood*.

